# Speeding Up Reachability Queries in Public Transport Networks Using Graph Partitioning

**DOI:** 10.1007/s10796-021-10164-2

**Published:** 2021-08-14

**Authors:** Bezaye Tesfaye, Nikolaus Augsten, Mateusz Pawlik, Michael H. Böhlen, Christian S. Jensen

**Affiliations:** 1 University of Salzburg, Salzburg, Austria; 2 University of Zurich, Zürich, Switzerland; 3 Aalborg University, Aalborg, Denmark

**Keywords:** Reachability queries, Public transport networks, Temporal graphs, Spatial network databases

## Abstract

Computing path queries such as the shortest path in public transport networks is challenging because the path costs between nodes change over time. A reachability query from a node at a given start time on such a network retrieves all points of interest (POIs) that are reachable within a given cost budget. Reachability queries are essential building blocks in many applications, for example, group recommendations, ranking spatial queries, or geomarketing. We propose an efficient solution for reachability queries in public transport networks. Currently, there are two options to solve reachability queries. (1) Execute a modified version of Dijkstra’s algorithm that supports time-dependent edge traversal costs; this solution is slow since it must expand edge by edge and does not use an index. (2) Issue a separate path query for each single POI, i.e., a single reachability query requires answering many path queries. None of these solutions scales to large networks with many POIs. We propose a novel and lightweight reachability index. The key idea is to partition the network into cells. Then, in contrast to other approaches, we expand the network cell by cell. Empirical evaluations on synthetic and real-world networks confirm the efficiency and the effectiveness of our index-based reachability query solution.

## Introduction

We study the problem of scalable and efficient reachability querying in public transport networks. A reachability query retrieves all points of interest (POIs) reachable from a given query node at a specific start time within a given time budget. The start time is required since the reachability result changes over time. Interesting applications of reachability queries include group recommendations, ranking spatial queries, urban planning, and geomarketing. We present two examples.

Consider a platform that recommends events to a group of people such that the group members like to attend the event together (Amer-Yahia et al., 2009; Jameson & Smyth, 2007). Group members are query nodes and events are POIs. When the group is given, the events must be evaluated by various criteria to optimize the benefit to the group. One important aspect is the location of the event relative to the group members. The start time and the travel time budget to reach an event may differ for each member. Events too far away are unlikely to be successful. A single recommendation comprises multiple reachability queries, one for each group member.

Another example is a real estate website that ranks properties (query nodes) according to user preferences. The users may customize reachability criteria for different POIs (e.g., school, working place, train station). Thereby, the time budget for individual types of POIs may vary: a user may be willing to commute to work for an hour, while a school must be nearby. Ranking the results of a single user query requires the computation of multiple reachability queries: one for each property and parameter setting.

To support such applications, reachability queries must be computed efficiently. Achieving this goal in public transport networks is tricky since the shortest path between two nodes depends on the start time, and the time to traverse a path may vary greatly across time. In a public transport network, stations are nodes, and connections between stations are edges between nodes. An edge can only be traversed at specific points in time as given by a schedule. Therefore, computing an index for public transport networks is more complex than for networks with constant edge-traversal costs or networks in which an edge can be traversed at any time (like pedestrian networks or road networks).

### *Example 1*

**Fig. 1:**
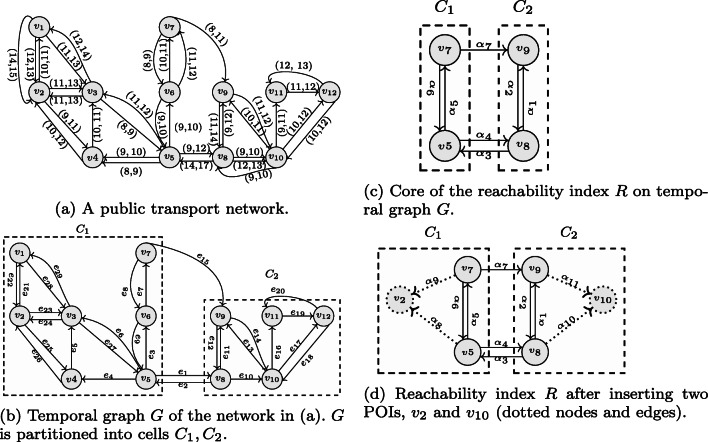
Temporal graph of public transport network and reachability index

Consider, the public transport network in Fig. 1a. The nodes *v*_1_, *v*_2_, …,*v*_12_ represent stations, and the directed edges represent connections between the stations. Each connection has a pair (*t*_*d*_, *t*_*a*_) of departure and arrival times. For example, there is a connection leaving *v*_4_ at time 10 and arriving at *v*_3_ at time 11. The traversal cost between nodes is expressed in terms of time units. The cost of traversing the edge (*v*_4_, *v*_3_) at time 9 is 2, since we have a waiting time in addition to the edge traversal time. The shortest path from *v*_10_ to *v*_11_ at start time *t*_*s*_ = 9 has cost 2 (edge (*v*_10_, *v*_11_)), while at *t*_*s*_ = 10, the cost of the shortest path is 3 (edges (*v*_10_, *v*_12_), (*v*_12_, *v*_11_)). At start time *t*_*s*_ = 9, the nodes {*v*_8_, *v*_9_, *v*_11_} are reachable from *v*_10_ with budget *Δt* = 2; at *t*_*s*_ = 10 with the same budget, we can reach the nodes {*v*_9_, *v*_12_}.

The state of the art in answering reachability queries in public transport networks includes two approaches. The first is based on a temporal version of Dijkstra’s algorithm (Dijkstra, 1959) that expands in the network until the budget is exhausted. Algorithms following this approach compute a so-called isochrone (the reachable region) and intersect it with the set of POIs (Bauer et al., 2008; Gamper et al., 2011). Since all edges in the isochrone must be expanded, these algorithms do not scale to large networks. The second approach translates a single reachability query into a set of path queries (e.g., shortest path or earliest-arrival path (Seufert et al., 2013; Wang et al., 2015; Wu et al., 2016)), one for each POI. Path queries require heavy index structures and do not scale to large numbers of POIs.

We propose an index-based technique for reachability queries in public transport networks. Instead of expanding edge by edge, in a precomputation step, we partition the network into cells and construct a novel reachability index. At query time, the index is used to expand cell by cell. Each cell covers a region of the network and all POIs in that region. The precomputation effort for a specific cell is independent of the other cells such that the index scales to large networks. The index is small, even smaller than the original graph for some inputs. This paper extends our preliminary results in Tesfaye et al. (2020) with an evaluation of different partitioning techniques. The idea of partitioning a public transport network to index reachability queries was first published in Tesfaye and Augsten (2016), but the proposed index is not exact and the quality of the approximation was not evaluated.

The rest of the paper is structured as follows. In Section 2, we define the problem, and we give an overview of our solution in Section 3. We introduce our reachability index in Section 4 and discuss query processing using the index in Section 5. In Section 6, we review related work. In Section 7 we investigate experimentally the performance of our solution. We conclude in Section 8.

## Preliminaries and Problem Definition

In a public transport network, stations are nodes and connections are edges. A connection has a departure time *t*_*d*_ and an arrival time *t*_*a*_, *t*_*d*_ < *t*_*a*_. We assume periodic schedules as is typically the case in public transport networks, e.g., schedules repeat daily or weekly.

A *temporal graph* *G* = (*V*, *E*, *c*) is a directed graph with vertices *V*, edges $E\subseteq V\times V$, and a time-dependent cost function *c*(*e*, *t*), $c:E\times \mathbb {R}\rightarrow \mathbb {R}_{\geq 0}$ that captures the time cost of traversing edge *e* starting at time *t*. We represent public transport networks as temporal graphs with a specific cost function, which we derive from the schedule. Each station is a node in the graph, and there is an edge from node *u* to node *v* iff there is a direct connection (i.e., there are no intermediate stops) from the station of *u* to the station of *v*. The cost function is periodic with period *π*, i.e., *c*(*e*, *t*) = *c*(*e*, *t* + *π*) and piecewise linear; all linear pieces have slope *k* = − 1; the cost function is not continuous; all discontinuities are at departure times of some connections. The time cost of taking a connection *s*_*i*_ = (*t*_*d*_, *t*_*a*_) to traverse edge *e* in the period (*t*_*d*_ −*π*, *t*_*d*_] is *σ*(*s*_*i*_, *t*) = *t*_*a*_ − *t*. If there are multiple connections *S*_*e*_ = {*s*_1_, *s*_2_,…,*s*_*i*_} on edge *e*, the cost of traversing edge *e* departing at time *t* is the minimum cost over all the individual connections at time *t*, $c(e,t)=\min \limits _{s\in S_{e}}\{\sigma (s,t)\}$. Our cost function is *consistent*, i.e., for any edge *e* ∈ *E* and all start times *t*_1_ ≤ *t*_2_: *t*_1_ + *c*(*e*, *t*_1_) ≤ *t*_2_ + *c*(*e*, *t*_2_). Intuitively, in a consistent cost function, it never pays off to skip a connection in order to wait for a later and faster connection. Consistency is required for the use of Dijkstra’s shortest-path algorithm (Kaufmann & Smith, 1993).

### *Example 2*

**Fig. 2:**
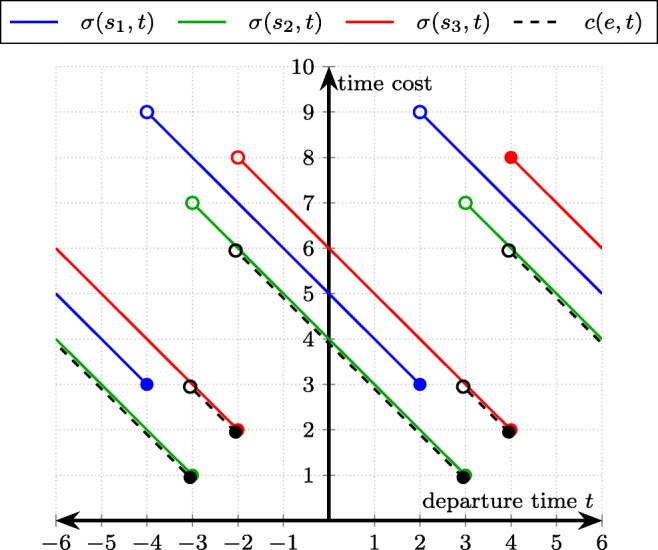
Example of cost function *c*(*e*, *t*) of traversing edge *e* starting at time *t* derived from three connections *s*_1_, *s*_2_, and *s*_3_ (period *π* = 6)

Consider an edge *e* with three connections *s*_1_ = (2,5), *s*_2_ = (3,4), *s*_3_ = (4,6) and a period of *π* = 6. Figure 2 shows the time costs (y-axis) of taking the individual connections to traverse edge *e* starting at time *t* (x-axis): *σ*(*s*_1_, *t*) = 5 − *t*, *t* ∈ (− 4,2] (blue), *σ*(*s*_2_, *t*) = 4 − *t*, *t* ∈ (− 3,3] (green), *σ*(*s*_3_, *t*) = 6 − *t*, *t* ∈ (− 2,4] (red). The overall cost function $c(e,t)=\min \limits \{\sigma (s_{1},t),\sigma (s_{2},t),\sigma (s_{3},t)\}$ is illustrated with black dashed lines. For example, *c*(*e*,2) = 2 (take connection *s*_2_ at cost *σ*(*s*_2_,2) = 4 − 2 = 2: depart at time 2, wait for 1 unit and then travel for 1 unit), *c*(*e*,4) = 2 (depart at time 4, no waiting, take connection *s*_3_ with cost *σ*(*s*_3_,4) = 6 − 4 = 2). Note that the connection *s*_1_ does not contribute to the cost function *c* because it departs earlier and arrives later than the connection *s*_2_. Thus, *s*_1_ is obsolete and $c(e,t)=\min \limits \{\sigma (s_{2},t),\sigma (s_{3},t)\}$ holds, making *c* consistent. In practice, connections like *s*_1_, if they exist, are removed from the schedule.

A *path* *p* from *u* to *v* in a temporal graph *G* = (*V*, *E*, *c*) is a sequence of edges *p* = 〈*e*_1_, *e*_2_,…,*e*_*n*_〉 such that *e*_*i*_ ∈ *E*, *e*_*i*_ = (*w*_*i*− 1_, *w*_*i*_), *w*_0_ = *u*, and *w*_*n*_ = *v*; *P*(*u*, *v*) is the *set of all paths* from node *u* to node *v*. The cost of a path is the fastest time to traverse the path at a given start time. Due to the consistency property of our cost function, the path cost is the sum of all edge costs. The *cost of path* $p=\langle e_{1},e_{2},\dots ,e_{n} \rangle $ at time *t*, *λ*(*p*, *t*), is the cost sum of all edges in *p*: $\lambda (p,t)={\sum }_{1\le i\le n}c(e_{i},t_{i})$, where *t*_1_ = *t* and *t*_*i*_ = *t*_*i*− 1_ + *c*(*e*_*i*− 1_, *t*_*i*− 1_) for *i* > 1. The *shortest-path cost* from node *u* to node *v* at time *t* is the minimum cost of any path from *u* to *v*, $sp(u,v,t)=\min \limits _{p\in P(u,v)}\{\lambda (p,t)\}$. A path with the minimum cost is called the *shortest path*. A node *v* is *reachable* from a node *u* at time *t* within budget *Δt* iff there is a path *p* ∈ *P*(*u*, *v*) such that the cost of *p* at time *t* is no larger than *Δt*, i.e., *λ*(*p*, *t*) ≤*Δt*.

### **Definition 1**

*Reachability query.* Given a temporal graph *G* = (*V*, *E*, *c*) with points of interst $POI \subseteq V$, the *reachability query* *RQ*(*u*, *t*, *Δt*) returns all points of interest reachable from node *u* at time *t* within budget *Δt*, i.e,

$$ RQ(u,t,{{\varDelta}} t) = \{v\in POI \mid \exists p\in P(u,v), \lambda(p,t)\le{{\varDelta}} t\} $$

### Problem definition

The goal of this work is to develop an efficient index-based solution for reachability queries that scales to large temporal graphs.

## Solution Overview

We propose a novel index structure, the *reachability index*, to answer reachability queries. We introduce a bulk loading technique for our index, provide access methods for answering reachability queries, and discuss the incremental insertion and deletion of POIs in the index.

The reachability index is built in a precomputation step. To construct the index, we partition the temporal graph into disjoint cells. Any such partitioning yields correct results. The choice of cells, however, affects the effectiveness of the index. We define requirements for a good partitioning and propose a suitable partitioning technique.

The index is a temporal graph that contains only those nodes of the original graph that are POIs or directly connect different cells, called *border nodes*. Each POI belongs to a cell. POIs can be inserted into and deleted from the index at any time; the update cost is low and depends on a single cell. The index consists of the original edges between border nodes of neighboring cells and new edges between the border nodes within a cell. Further, an edge between each POI and the border nodes in its cell is introduced. The edge costs are the costs of shortest paths between the respective nodes in the original graph.

A high number of border nodes per cell increases the index size. Each POI adds as many edges to the index as there are border nodes in its cell.

A search query traverses the index cell by cell. The border nodes are used to cross cells and to reach neighboring cells. For each border node, we verify if any of the POIs in that cell is reachable.

## The Reachability Index

The reachability index *R* is a temporal graph that is constructed from the original graph *G* as follows:

1. *Graph partitioning.* The nodes of graph *G* are split into disjoint *cells*. At query time, instead of expanding edge by edge in *G*, we expand cell by cell in the index.
2. *Constructing the index core.* Based on the graph partitioning, we insert the so-called *border nodes* and edges into the initially empty index. This index core never changes.
3. *Computing the index cost function.* The edge cost is computed as a shortest-path cost for each departure time from a source node to a destination node.
4. *Inserting POIs.* Inserting a POI into a cell adds a new node and an edge to each border node of the cell. POIs that are not *border nodes* can be inserted and deleted dynamically without modifying the rest of the index.

We detail each step of the index construction next. Additionally, we discuss the factors that affect the size of the reachability index and present a compaction technique to reduce the number of connections.

### Graph Partitioning

We partition the nodes of a temporal graph *G* = (*V*, *E*, *c*) into a set of disjoint cells $C=\{C_{1},C_{2},\dots ,C_{n}\}$, such that each node of *G* belongs to exactly one cell *C*_*i*_, i.e., *C*_*i*_ ∩ *C*_*j*_ = *∅* for any pair of cells with *i*≠*j*, and $\bigcup _{1\le i\le n}C_{i}=V$. Each disconnected component of the graph that is supposed to be indexed^1^ should be partitioned into at least two cells. The nodes of a cell *C*_*i*_ that have an adjacent node in a cell different from *C*_*i*_ are *border nodes*, *B*_*i*_: A node *v* ∈ *C*_*i*_ is a border node, *v* ∈ *B*_*i*_, if it has an edge to or from another cell, i.e., there is a node *w* ∈ *V*, *w*∉*C*_*i*_, and an edge (*v*, *w*) ∈ *E* or an edge (*w*, *v*) ∈ *E*. For example, the temporal graph *G* in Fig. 1b of our example public transport network (Fig. 1a) is partitioned into two cells (dashed boxes): *C*_1_ with border nodes *v*_5_ and *v*_7_, and *C*_2_ with border nodes *v*_8_ and *v*_9_.

The cells define the structure of the reachability index. The index will be expanded cell by cell to answer reachability queries. A good partitioning should satisfy the following properties:

1. *Well connected inside.* A cell comprises highly-linked nodes with many edges and connections inside the cell.
2. *Loosely connected outside.* The number of border nodes per cell is small.
3. *Large distance between cells.* Crossing cell borders is expensive: the number of connections between cells is small and their cost is high.

Finding a good partitioning that satisfies our requirements is not straightforward. We identify two relevant approaches in literature: *community detection* (Blondel et al., 2008; Newman & Girvan, 2004; Traag et al., 2019) and *minimum edge-cut partitioning* (Karypis & Kumar, 1998a; 1998b). Both approaches strive to minimize the edges connecting different partitions. An additional objective of the minimum edge-cut partitioning is to produce partitions of balanced size. Community detection algorithms automatically adjust the number of partitions such that the resulting partitions are densely connected inside. For the minimum edge-cut partitioning the number *k* of partitions is a user-defined parameter. Both approaches allow for edge weights that increase the cost for cutting an edge; we choose the number of connections on an edge to be its weight.

We assess how well do community detection and minimum edge-cut partitioning suit our scenario. In this section, we revisit the commonly used algorithms and thoroughly evaluate their impact on our solution in Section 7.

#### Community Detection

In contrast to fixing the number of partitions upfront, community detection aims at identifying naturally occurring densely connected subgraphs. One of the best-known objective functions for community detection is *modularity* (Newman & Girvan, 2004). Modularity is the difference between the edges in the identified communities and the expected number of edges in communities of an equivalent network with edges distributed at random. Optimizing modularity, i.e., finding communities that maximize modularity, is NP-hard. We describe two approximations: Louvain (Blondel et al., 2008), and its recent improvement Leiden (Traag et al., 2019).

##### Louvain

by Blondel et al. (2008) is a two-phase approach. Initially, each node forms its own community. In the first phase, nodes are moved to neighboring communities such that the modularity increases. The result is the input to the second phase, where an aggregated network is created. Each node in the aggregated network represents a community detected in the first phase. The two phases are repeated until modularity cannot be increased any further.

Louvain produces partitions with the following guarantees: no communities can be merged and no nodes can be moved between communities to further improve modularity (Traag et al., 2019).

##### Leiden

by Traag et al. (2019) is an improvement of Louvain. Traag et al. observe that Louvain may result in poorly connected or even disconnected communities, which is not desired. Leiden adds an additional phase to Louvain. Initially, each node is in its community. In the first phase, similarly to Louvain, Leiden moves nodes to neighboring communities. However, unlike Louvain that visits each node, Leiden uses an improvement, called *fast local move*, to visit only those nodes whose neighborhood has changed. In the second phase, called refinement, Leiden finds sub-communities within each community resulted from the first phase. Each of the sub-communities will form their own community. The refinement phase is used to avoid poorly connected and disconnected communities. In the third phase, unlike Louvain, Leiden uses the refined communities to aggregate the network.

Leiden provides two new guarantees in addition to the guarantees provided by Louvain: communities are well connected, and individual nodes are well connected to their communities.

#### Minimum Edge-Cut Partitioning

The minimum edge-cut partitioning computes *k* partitions such that: (a) the sizes of the partitions are balanced, (b) the number of edges that connect nodes from different partitions (the so-called *edge-cut*) is minimized. Finding the minimum edge-cut is NP-hard. Efficient approximate methods resulting in high-quality partitions follow a multilevel partitioning paradigm. For our purpose we chose the multilevel k-way partitioning (Karypis and Kumar, 1998b) from the commonly used METIS framework (Karypis & Kumar, 1998a).

The multilevel k-way partitioning (Karypis & Kumar, 1998b) is a three-phase approach, consisting of *coarsening*, *partitioning*, and *uncoarsening*. In *coarsening phase* an input graph is reduced by collapsing nodes and edges iteratively. In each step, the graph from the previous step is reduced further. The coarsening phase stops when the coarsest graph has a small number of nodes, or the reduction between two consecutive coarser graphs is too small. The *partitioning phase* splits the coarsest graph into *k* partitions. The *uncoarsening phase* projects the partitioned graph back to the original input by unfolding nodes and edges. To do so, it follows the backward sequence of coarsened graphs until all nodes and edges are unfolded. During uncoarsening, a refinement technique is applied to decrease the edge-cut. Various algorithms have been proposed for each of the phases; Karypis and Kumar (1998b) thoroughly evaluate the options.

In our scenario, the number *k* of desired partitions is not known up front. This parameter is hard to guess. Since Leiden and Louvain do not require this input parameter, the number of partitions produced by these algorithms could serve as a guideline. This is the approach that we take in our empirical evaluation.

### Constructing the Index Core

Given a temporal graph *G* = (*V*, *E*, *c*) and a partitioning *C* of *G*, we construct the core of our reachability index. The index core is independent of POIs and never changes for a given partitioning. The reachability index is a temporal graph *R* = (*V*_*R*_, *E*_*R*_, *c*_*R*_) with nodes *V*_*R*_ ⊂ *V*, edges $E_{R}\subseteq V_{R}\times V_{R}$, and cost function *c*_*R*_(*e*, *t*) on the edges *e* ∈ *E*_*R*_. For an edge *e* = (*u*, *v*) ∈ *E*_*R*_, *c*_*R*_ is defined as the shortest-path cost from *u* to *v* at time *t* in *G*, i.e., *c*_*R*_(*e*, *t*) = *sp*(*u*, *v*, *t*).

*Index nodes.* For each cell *C*_*i*_ ∈ *C*, we insert all its *border nodes* *B*_*i*_ into the node set *V*_*R*_ of the index. Thus, the nodes of the index $V_{R} = \bigcup _{1\le i\le |C|} B_{i}$. Figure 1c shows the index core of the temporal graph (Fig. 1b) with cells *C*_1_ = {*v*_5_, *v*_7_} and *C*_2_ = {*v*_8_, *v*_9_}.

#### Index edges

The edges of the index core are *E*_*R*_ = *BB* ∪*BC*. *BB* is the set of all edges between border nodes of neighboring cells. For each edge (*u*, *v*) ∈ *E* between two border nodes of different cells in *C*, *u* ∈ *C*_*i*_, *v* ∈ *C*_*j*_, *i*≠*j*, we insert a new edge between the respective nodes into the index, *E*_*R*_ = *E*_*R*_ ∪{(*u*, *v*)}. *BC* is the set of all edges between pairs of border nodes within a cell. For each pair *u*, *v* ∈ *B*_*i*_, we insert two new edges (*u*, *v*) and (*v*, *u*) into the index, *E*_*R*_ = *E*_*R*_ ∪{(*u*, *v*),(*v*, *u*)}. For example, *BB* = {*α*_3_, *α*_4_, *α*_7_} and *BC* = {*α*_1_, *α*_2_, *α*_5_, *α*_6_} in Fig. 1c.

### Points of Interest

POIs can be inserted and deleted at any time, also after index construction. This is beneficial because POIs may change over time. A POI *v* ∈ *V* may be any node in the original temporal graph. If *v* is a border node, no action is required because such a node is in the index core already. Otherwise, similarly to border nodes, inserting *v* into the index involves three steps. (1) We add *v* to the index nodes (*V*_*R*_ = *V*_*R*_ ∪{*v*}). (2) We add a new edge from each border node of *v*^′^*s* cell to *v* (we call such edges BP edges). (3) The cost function based on shortest paths (same procedure as for all other edges) is computed. Deleting a POI from the index removes the POI node and all its incoming edges. For example, consider inserting two POIs, *v*_2_, *v*_10_, into the index in Fig. 1d. We add edges *BP* = {*α*_8_, *α*_9_, *α*_10_, *α*_11_}.

### Computing the Index Cost Function

The cost function *c*_*R*_ of an edge *e* = (*u*, *v*) ∈ *E*_*R*_ in index *R* is defined as the shortest-path cost from *u* to *v* at time *t* in graph *G*, i.e., *c*_*R*_(*e*, *t*) = *sp*(*u*, *v*, *t*). For computing the values of the cost function *c*_*R*_, we execute Dijkstra’s single-source shortest-path algorithm once for every border node *b* ∈ *B*_*i*_ and every departure time at *b*. The expansion stops when all other border nodes in the cell and all direct neighbors of *b* (i.e., nodes reachable from *b* via a *BB* edge) are visited. Since the cells are small compared to the overall graph, typically only a small number of nodes needs to be considered for each execution of Dijkstra’s algorithm. *BC* and *BP* edges may connect nodes that are not reachable in the original temporal graph. If a node is not reached during one of the shortest-path computations, we assign infinite cost to the respective edges.

#### *Example 3*

Cost examples for the index core in Fig. 1c are: *c*_*R*_(*α*_3_,14) = 3, *c*_*R*_(*α*_1_,9) = 2, *c*_*R*_(*α*_5_,9) = 2. In Fig. 1d, the costs for the edges of type *BP* are *c*_*R*_(*α*_8_,9) = 3, *c*_*R*_(*α*_9_,8) = 7, *c*_*R*_(*α*_10_,9) = 1, and *c*_*R*_(*α*_11_,11) = 1.

### Index Size

The index consists of border nodes and POIs. Thus, the number of index nodes is at most the number of nodes in the temporal graph. We introduce three types of edges into the index. *BB* edges connect border nodes between different cells, and they are a subset of the temporal graph edges. *BC* edges connect border nodes in a single cell, and their cardinality is at most quadratic in the number of border nodes. Each POI adds as many *BP* edges as border nodes in a cell. The numbers of *BC* and *BP* edges depend only on the subset of temporal graph nodes that are in a single cell. The numbers do not depend on the graph size. In sparse graphs, where many nodes have only a few edges, the reachability index may grow larger than the temporal graph: we can remove only a small number of original edges but need to insert new *BC* and *BP* edges.

Each edge has as many edge cost values as there are departure times from a node. The edge costs are computed for each single cell in isolation, making parallel computation possible. In particular, the edge cost of a specific border node at a specific departure time is independent of all other edge costs.

### Index Compaction

The index size, as well as the size of the temporal graph, is dominated by the size of the schedule, i.e., the number of edge connections. After computing the edge costs in the index, we observe that many different departure times have the same arrival time at the destination. It is enough to keep only one connection per arrival time, namely the one with the maximum departure time. We leverage that and compact the index by reducing the number of connections as follows. Consider an edge *e*(*u*, *v*) ∈ *E*_*R*_ and set *S* of departure–arrival connection pairs (*d*, *a*) on that edge. We compact *S* to $S^{\prime }\subseteq S$, such that $S^{\prime }=\{ (d,a)\in S : \nexists _{(d_{i},a_{i})\in S} a_{i}=a \wedge d_{i}>d \}$. Experiments show that this compaction technique is highly effective and reduces the index size by up to 74% (cf. Section 7). For example, the set of all connections on edge *α*_8_ in Fig. 1d, {(8,12),(9,12),(11,15)}, is compacted into {(9,12),(11,15)}.

## Answering Reachability Queries

The core idea of our reachability algorithm is to expand cell by cell rather than edge by edge. The *BB* edges between border nodes of different cells allow us to expand to the neighboring cells; the *BC* edges between border nodes of the same cell reflect the time to cross a cell; the direct *BP* edges from border nodes to POIs allow for a quick evaluation of which POIs can be reached. In addition, we discuss a heuristic to avoid unnecessary edge expansions and processing of query nodes that are non-border nodes.

### The reachability algorithm

Algorithm 1 takes as an input the reachability index *R* = (*V*_*R*_, *E*_*R*_, *c*_*R*_), query node *q*, start time *t*_*s*_, and the cost budget *Δt*. The expansion proceeds like in Dijkstra’s algorithm and returns the set *N* of reachable POIs in *R*. Nodes and their costs from *q* are stored in a min-heap *M* initialized to *M*[*q*] = 0, and $M[v]=\infty $ for all other nodes *v* (line 1). The closest node *v* to *q* is popped from the min-heap (line 4), and the costs for nodes adjacent to *v* are updated if smaller (lines 9–11). To retrieve the correct edge cost, we do a binary search in the list of edge costs sorted by departure time (line 9). Each node is traversed only once. The algorithm terminates when no more nodes with cost lower than the budget are in the heap (line 5). Consider the reachability index in Fig. 1d. Here, *RQ*(*R*, *v*_5_,8,6) = {*v*_2_, *v*_10_} because *sp*(*v*_5_, *v*_2_, *t*) = 4 (through *α*_8_) and *sp*(*v*_5_, *v*_10_, *t*) = 5 (through *α*_4_ and *α*_10_). *RQ*(*R*, *v*_5_,6,6) = {*v*_2_} because *sp*(*v*_5_, *v*_2_, *t*) = 6 (through *α*_8_) but *sp*(*v*_5_, *v*_10_, *t*) = 7 (through *α*_4_ and *α*_10_). 
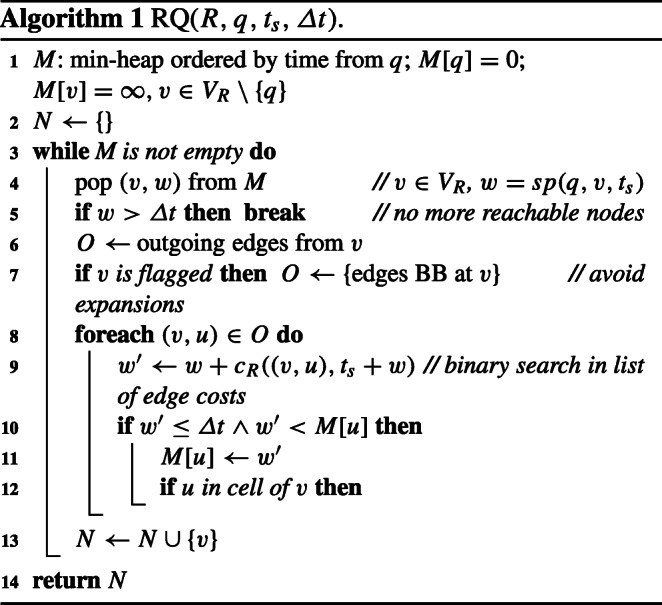


### Avoiding unnecessary expansions

Regarding the edges within a cell, we observe the following. Consider Algorithm 1 processing a border node *b* of a cell *C*_*i*_. Then, the costs of the other nodes, *v*_*j*_ ∈ *C*_*i*_, are updated w.r.t. the cost of reaching them from *b*. When we pop a node *v*_*j*_ in a later round, and if *v*_*j*_ was last updated by *b*, there is no point in following the edges from *v*_*j*_ to the other nodes in the cell. The cost of accessing the other nodes in the cell through *v*_*j*_ cannot be smaller than the cost of accessing these nodes directly from *b* since all edge costs are shortest paths. If, however, *v*_*j*_ was updated through an edge from a neighboring cell, the edges to the other nodes in the cell need to be followed. We exploit this observation to avoid following edges inside a cell that cannot lead to an update and thus do not affect the solution. We flag the nodes whenever their cost was updated by processing a node from within a cell, and we remove the flag, otherwise (line 12). The outgoing edges that must be expanded are selected based on the flag (line 7).

Note that the number of edges within a cell is quadratic in the number of border nodes of that cell. Thanks to the use of flags we avoid unnecessary expansions. In particular, if the cheapest way to reach all nodes in a cell is through *k* border nodes, we only expand *k*(*w* − 1) edges per cell, where *w* is the number of all border nodes and POIs in a cell. The value of *k* is expected to be small and will often be 1 (i.e., the shortest path from a query node *q* to all nodes in the cell crosses the border node that is closest to *q*).

### Non-border query nodes

The reachability index does not contain all nodes of the original graph. If the query node *q* in cell *C*_*i*_ is not a border node, the algorithm starts the expansion from *q* in the temporal graph. All POIs reached in cell *C*_*i*_ are part of the result. Once a border node $b^{\prime }\in B_{i}$ is reached, the expansion continues in the index at time $t_{s}+\textit {sp}(q,b^{\prime },t_{s})$.

### Correctness

We show that the shortest-path costs in the index and the original temporal graph are identical. Let *u*, *w* ∈ *V*_*R*_ be two index nodes and *p* = 〈(*v*_0_, *v*_1_),(*v*_1_, *v*_2_),…,(*v*_*n*− 1_, *v*_*n*_)〉 be the corresponding shortest path in the temporal graph, i.e., *u* = *v*_0_, *w* = *v*_*n*_. If there is a direct edge between *u* and *w* in the index, the shortest-path cost is the cost of that edge: this cost is precomputed using Dijkstra’s algorithm for each departure time in the original temporal graph; since our cost function is consistent (cf. Section 2), the edge cost is correct (Kaufmann & Smith, 1993). Otherwise, *u* and *w* are not in the same cell (all nodes in a cell are connected with an edge). So, there must be a path along index nodes $u_{1}, u_{2}, {\ldots } u_{k} \subseteq v_{1}, {\ldots } v_{n-1}$ that are all on path *p* since cells can be exited only through border nodes. We show that the cost of the index path is indeed the shortest path. Assume a node *u*_*i*_ exists such that *sp*(*v*_0_, *v*_*n*_, *t*) < *sp*(*u*, *u*_*i*_, *t*) + *sp*(*u*_*i*_, *w*, *t*) + *sp*(*u*, *u*_*i*_, *t*). On a path of length two, the costs of edges (*u*, *u*_1_) and (*u*_1_, *w*) are precomputed shortest-path costs, and they are therefore correct. The assumption, however, implies that one of the edge costs could be decreased, i.e., the assumption is incorrect. This argument can be extended edge by edge to paths of arbitrary length.

## Related Work

Shortest-path and reachability queries on road networks, i.e., graphs with constant edge cost, have been studied extensively. Unfortunately, these works cannot be applied readily to public transport networks (Bast, 2009). An evaluation by Bast et al. (Bast et al., 2016) shows a large performance gap between the two types of networks. This is due to the time-dependent edge costs of public transport networks, which makes the precomputation efforts of many algorithms infeasible.

Current solutions for public transport networks either rely on Dijkstra’s algorithm (Dijkstra, 1959) or require heavy precomputations. Dijkstra’s algorithm follows an expansion technique that visits edges in all possible direction until the target is reached. Dijkstra-based approaches include isochrone algorithms for multimodal networks (Bauer et al., 2008; Gamper et al., 2011). They expand from a query point using Dijkstra’s algorithm and compute a so-called isochrone, which is the reachable portion of the network at a given point in time. Since all edges in the isochrone must be expanded, this approach does not scale to large networks.

Many works fall into the category of labeling approaches. The earliest work, 2-hop labeling (Cohen et al., 2003), is designed for weighted graphs and is based on 2-hop covers of shortest paths. Recent works strive to decrease the index size and construction time (Cheng et al., 2013; Jin & Wang, 2013), which are bottlenecks of 2-hop labeling and prevent application to large graphs. Time Table Labeling (TTL) (Wang et al., 2015) and Top Chain (Wu et al., 2016) adapt 2-hop labeling to public transport networks; they support shortest-path and point-to-point reachability queries. In TTL, the main idea is to precompute label sets for each node *v* containing reachable nodes from and to *v*. Top Chain creates a directed acyclic graph (DAG), where each node represents a departure time, and decomposes the DAG to create the label sets. Creating label sets in both techniques requires high precomputation costs and large index sizes. To decrease the index size, Top Chain only stores *K* label sets, called chains. The index size of Top Chain for small *K* values is smaller than that of TTL, but there is no guarantee that the query results can be found using the index.

Non-labeling techniques include Scalable Transfer Patterns (Bast et al., 2016), Connection Scan Algorithm (CSA) (Strasser, 2016), and Contraction Hierarchy for Timetables (CHT) (Geisberger, 2010). Transfer Patterns require an expensive profile search from each node to find the optimal paths to all other nodes. CSA organizes a schedule as two sequences of edges. The first sequence contains sorted edges based on arrival times, and the second sequence sorts edges based on departure times. At query time, CSA scans the sequences in linear time to answer earliest arrival path queries. CHT organizes vertices in hierarchies and applies a contraction technique to reduce the graph size for query processing. SPs are precomputed by adding new edges to the graph, which are leveraged at query time. These approaches involve expensive precomputations or large index sizes, which limits their scalability.

To compute reachability queries as defined in this paper, all techniques based on point-to-point queries require the computation of shortest paths from a given query node to every POI, which does not scale to large number of POIs.

## Experiments

We experimentally evaluate our solution, *RQ* , and compare it to two competitors, a no-index solution, *NI* , and a fully-indexed solution, *SP* . We report on the index size and efficiency of the algorithms w.r.t. the number of expanded edges, which is the work that an algorithm has to do to find reachable nodes.

Our algorithm, *RQ* , partitions the input graph in order to build the index. We study the effect of different partitioning techniques discussed in Section 4.1 on the index structure and the performance of our *RQ* algorithm. We identify one partitioning technique to be used in conjunction with *RQ* .

### Implementation details

The algorithms are implemented in Python 3 and executed on a virtual machine with 32 cores and 117GB RAM, running Debian 10(buster). For the partitioning of the input graphs we use the following Python libraries: for Louvain python-louvain v0.13^2^, for Leiden leidenalg v0.8.2^3^, for multilevel k-way partitioning metis v2020.1^4^ with the underlying C METIS library v5.1.0^5^.

### Competitors

#### No-Index (NI)

The no-index solution, *NI* , operates on the original temporal graph and does not build an index. The reachability query is computed with a modified version of Dijkstra’s algorithm that supports our cost function (cf. Section 5).

#### Shortest-Path (SP)

The fully-indexed solution, *SP* , precomputes and stores all shortest paths from every node in the temporal graph to each POI at every departure time. *SP* represents the collection of works that index the shortest paths between pairs of nodes (cf. Section 6). In terms of lookups per shortest-path, *SP* is optimal since only a single lookup is required per shortest-path query. Other solutions for reachability queries based on indices for shortest-path queries cannot outperform *SP* in terms of lookups per query. Instead, these solutions trade in lookup performance to achieve a smaller memory footprint (which is high for *SP*). Therefore, the number of edge expansions performed by *SP* for reachability queries cannot improve by substituting *SP* by another shortest-path index.

### Datasets

**Fig. 3:**
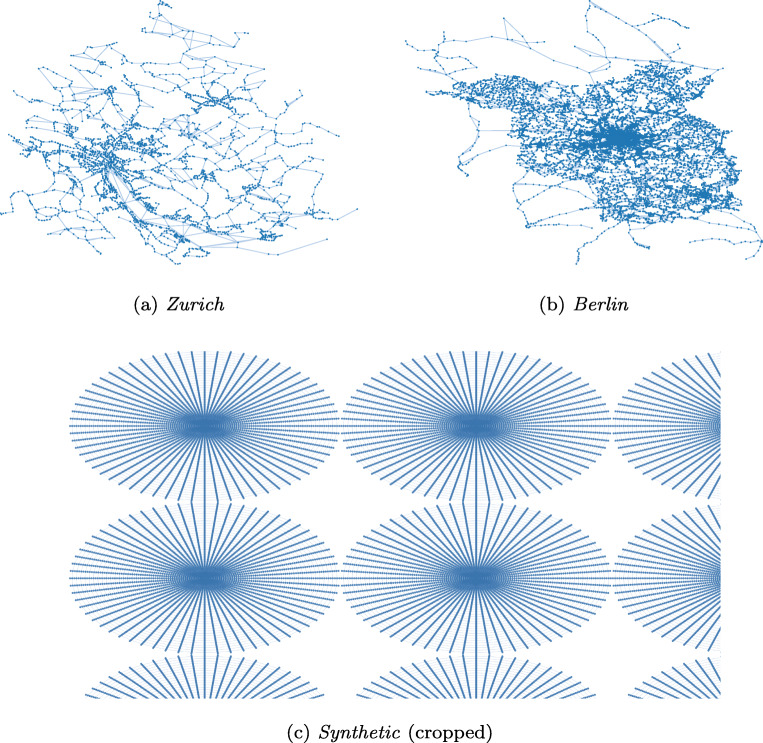
Structure of the different public transport networks

We use two real-world public transport networks represented as temporal graphs, Zurich and Berlin (2020), and one synthetic graph, *Synthetic*. *Zurich* and *Berlin* are obtained in GTFS format that is further processed. For these graphs, we chose all transport modes and all connections operating on Mondays. *Synthetic* is a 6 × 6 grid of equally-sized spider-web subgraphs. Each spider-web subgraph has one edge to every neighboring subgraph (to its left, right, top, and bottom). This graph simulates loosely connected cities that are densely connected inside. In such a case we would expect a good partitioning to assign nodes of each spider-web subgraph into a separate partition. Table 1 shows the statistics, where #Conn is the number of all connections (departure-arrival pairs) that can be used to cross an edge. Figure 3 visualizes the structure of our public transport networks.

**Table 1:** Dataset statistics

Dataset	#Nodes	#Edges	#Conn
Zurich	2,508	5,630	555,713
Berlin	12,984	34,791	1,348,070
Synthetic	145,188	433,272	31,042,468

### Effect of Partitioning

Our solution, *RQ* , will work with any partitioning of the input graph and compute correct results. However, we observe that the index structure and performance of query answering varies depending on the specific partitioning used to build the index (cf. Section 4.1).

We investigate the effect of different partitioning techniques on our solution *RQ* . In our analysis, we include two community detection algorithms, Louvain (Blondel et al., 2008) and Leiden (Traag et al., 2019), and the multilevel k-way partitioning (Karypis & Kumar, 1998b) from the METIS framework (Karypis & Kumar, 1998a) (denoted as METIS). In Louvain and Leiden, the so-called modularity of the partitioning is optimized to find good communities. To compute the modularity, a resolution and a weight between pairs of nodes needs to be specified. We use the default value 1 for the resolution and the number of connections as the edge weight: the more connections exist between two nodes, the better they are connected.

While Louvain and Leiden auto-detect the number of partitions, METIS requires the number of partitions as an input parameter. Defining a good number of partitions is not straightforward since this parameter inherently depends on the network structure. We evaluate METIS using the mean of the partition numbers detected by Louvain and Leiden when we compare to these algorithms in terms of index size and query performance.

#### Structure of the Partitioning

**Fig. 4:**
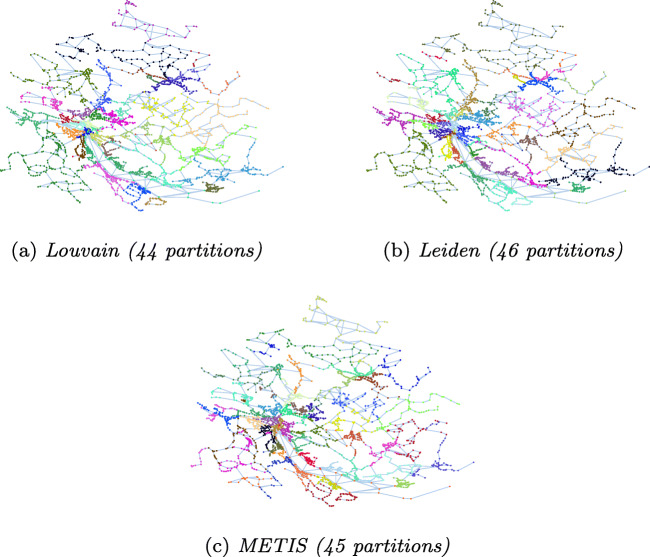
Partitioning of Zurich

**Fig. 5:**
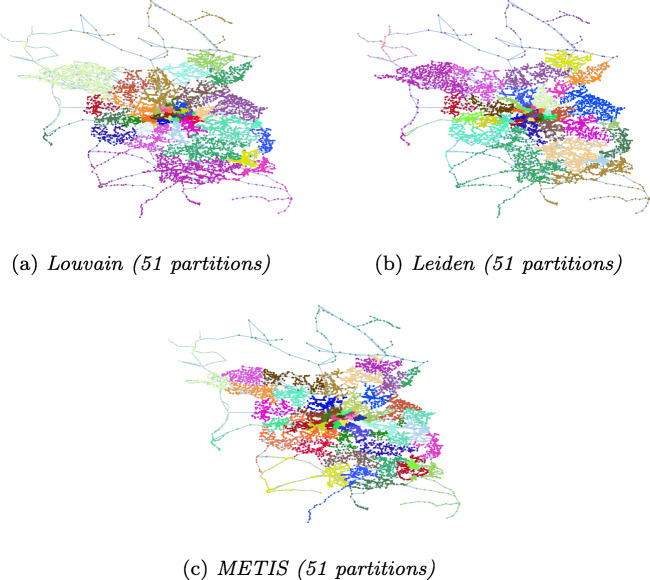
Partitioning of Berlin

**Fig. 6:**
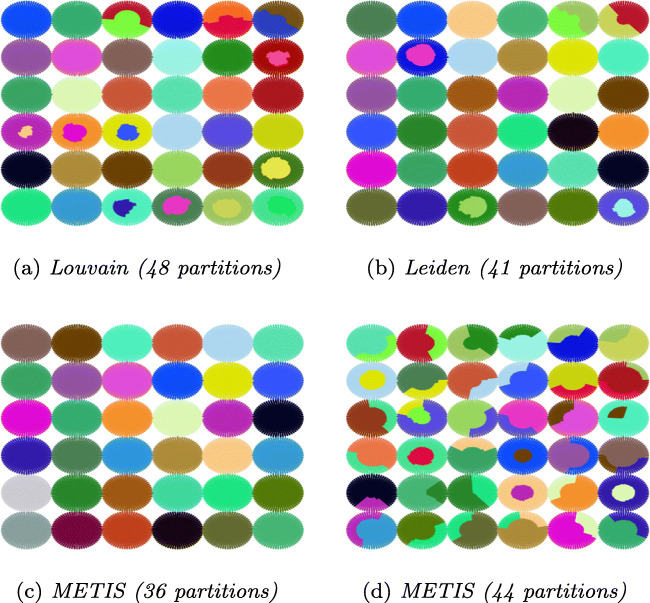
Partitioning of Synthetic (each partition is colored different)

Table 2 shows the statistics of partitions resulting from applying different techniques to our input graphs: number of partitions, partition sizes (average/min/max), number of border nodes (total sum, average/min/max per partition). We visualize the partitions for Zurich, Berlin and Synthetic graphs in Figs. 4, 5, and 6, respectively.

**Table 2:** Statistics for different partitioning techniques

Dataset	Partitioning					Border nodes			
	Algo.	#Part	avg	min	max	sum	avg	min	max
Zurich	Louvain	44	57	2	163	314	7	0	20
	Leiden	46	54.5	2	158	298	6.5	0	17
	METIS	45	55.7	54	57	439	9.8	1	20
Berlin	Louvain	51	254.6	2	924	1250	24.5	0	52
	Leiden	51	254.6	2	747	1138	22.3	0	43
	METIS	51	254.6	247	262	1439	28.2	9	53
Synthetic	Louvain	48	3024.8	1042	4037	1979	41.2	2	90
	Leiden	41	3541.2	1463	4036	904	22	2	84
	METIS	44	3299.7	3204	3398	4395	99.9	51	227
		36	4033	4031	4035	120	3.3	2	4

##### Number of Partitions

Louvain and Leiden auto-detect the number of partitions. The number of detected partitions is similar for both algorithms: Louvain detects 4*%* fewer partitions than Leiden for Zurich and 15*%* more partitoins for Synthetic; both algorithms produce the same number of partitions for Berlin. METIS requires the number of partitions as an input parameter. We choose the mean value of the partition numbers detected by Louvain and Leiden for METIS. For Synthetic, in addition, we provide METIS with the number of spider webs (6 × 6 = 36) that we used to construct the graph. The numbers in Table 2 show that METIS correctly identifies all spider webs when the number of partitions is well chosen, resulting in a low number of border nodes. With the number of partitions that we automatically detect using Louvain and Leiden (44 partitions), however, the performance of METIS significantly drops. The visualization of the partitions in Fig. 6 confirms this result.

##### Size of Partititions

**Fig. 7:**
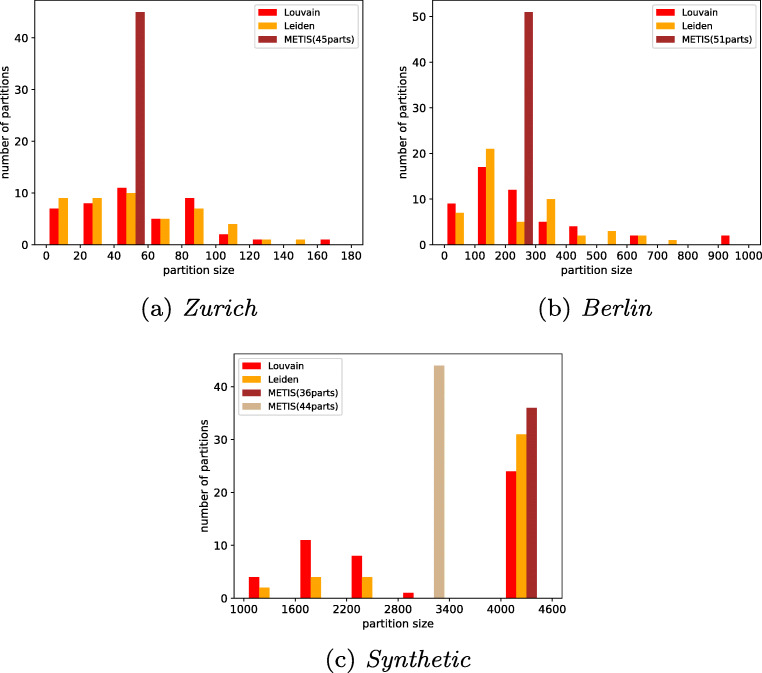
Distribution of nodes per partition

One of the optimization goals of METIS is to produce partitions well balanced in size, whereas Louvain and Leiden detect communities regardless of their size. This is confirmed by our results. The partitions of METIS are well balanced, while the community detection algorithms produce some very small and some very large partitions for the real world datasets. Figure 7 shows the distribution of nodes per partition for the three algorithms on Zurich, Berlin and Synthetic.

##### Number of Border Nodes

As discussed in Section 4.1, we strive for partitions that are well connected inside and loosely connected outside. A low number of border nodes indicates loose connections between partitions since edges between partitions can exist only between border nodes. For Zurich and Berlin, Leiden exhibits the lowest values for border nodes. We observe that for these datasets the good balance of partition sizes of METIS comes at the cost of more border nodes.

For the Synthetic graph, if METIS is given the optimal number of 36 partitions, all spider webs are detected and optimal partitions are produced, resulting in two border nodes for the spider webs in the four corners, three border nodes for the spider webs on the boundaries of the graph, and four border nodes for all other spider webs. With the auto-detected value of 44 partitions, METIS produces the largest number of border nodes for Synthetic. The reason is that METIS strives to balance the partition sizes also at the cost of more border nodes and border edges.

Zurich and Berlin include small disconnected components. Louvain and Leiden detect the small disconnected components and do not further partition them. This explains the zero values for the minimum number of border nodes in Table 2. METIS includes the disconnected components into larger partitions to balance their sizes.

#### Index Size and Structure

The border nodes resulting from a partitioning define the structure of our *RQ* index. Table 3 presents the index core size for different partitionings. The suffixes LO, LE, and ME*k* in the Algorithm column denote Louvain, Leiden, and METIS with *k* partitions, respectively. The values that increase the index size are the number of nodes (#Nodes) and edges (#Edges), and the number of connections (#Conn), which is the dominating factor. The total sum of these three values (#Total) is used to compare index sizes among the different partitionings.

**Table 3:** Index core size for different partitionings

Dataset	Algorithm	#Nodes	#Edges	#Conn	#Total
Zurich	RQ_LO	314	3,333	405,898	409,545
	RQ_LE	298	2,953	377,818	381,069
	RQ_ME45	439	5,131	436,281	441,851
Berlin	RQ_LO	1,250	38,286	2,229,584	2,269,120
	RQ_LE	1,138	30,124	1,936,377	1,967,639
	RQ_ME51	1,439	45,664	1, 976, 750	2,023,853
Synthetic	RQ_LO	1,979	150,840	13,263,713	13,416,532
	RQ_LE	904	63,622	5,833,430	5,897,956
	RQ_ME36	120	416	22,025	22,561
	RQ_ME44	4,395	598,896	35,192,991	35,796,282

Table 3 suggests that the most promising partitioning is Leiden, which results in the smallest overall index core size (#Total). An exception is Synthetic if we provide METIS with the optimal number of 36 partitions: the resulting index core is much smaller than the index core for the other partitionings. With the auto-detected number of 44 partitions, however, METIS produces the largest index core.

To evaluate our *RQ* index size after additions of POIs, we randomly pick 5% of the input graph nodes to be POIs and we built our index for different partitionings across five different POI samples. Table 4 shows average (avg) and standard deviation (*σ*, rounded values) of the counts that affect the index size (number of nodes, edges, and connections). In Zurich and Berlin, the average number of nodes and edges using Leiden is the smallest. On the Synthetic dataset, METIS with 36 partitions outperforms all the other partitioning algorithms for all values. The number of nodes varies across the samples due to a varying number of POIs that are *border nodes*. The standard deviation is below 2% for any of the values, indicating that the particular choice of POIs does not have a large effect on the index size.

**Table 4:** Index size for different partitionings given multiple samples of POIs

Dataset	Algorithm	#Nodes		#Edges		#Conn	
	Algorithm	avg	*σ*	avg	*σ*	avg	*σ*
Zurich	RQ_LO	421	4	4,173	47	456,913	7,206
	RQ_LE	406	2	3,733	46	425,621	11,207
	RQ_ME45	542	2	6,102	35	494,138	8,437
Berlin	RQ_LO	1,834	8	54,988	283	2,385,623	7,103
	RQ_LE	1,732	5	44,643	161	2,717,979	13,747
	RQ_ME51	2,022	10	61,893	199	2,539,917	14,743
Synthetic	RQ_LO	9,135	9	350,070	2,811	28,873,351	259,401
	RQ_LE	8,122	7	163,449	1,982	13,646,302	173,456
	RQ_ME36	7,375	2	24,594	61	1,420,407	7,379
	RQ_ME44	11,423	18	1,281,986	5,555	78,219,714	205,325

#### Query Performance.

To measure the effect of different partitioning techniques on the querying performance, we count the number of edges that *RQ* must expand for answering a reachability query. We compare the indices constructed over the partitioning of Louvain, Leiden, and METIS.

In this experiment, we use the *RQ* index constructed for one of the POI samples discussed in Section 7.4.2. All query nodes are border nodes. Since the different partitionings produce different border nodes, we pick all border nodes that exist in all partitionings as query nodes. For each query node, we executed a total of 10 different reachability queries varying the starting time (8:00, 12:00, 16:00, 18:00, 22:00) and time budget (60 and 120 minutes).

**Fig. 8:**
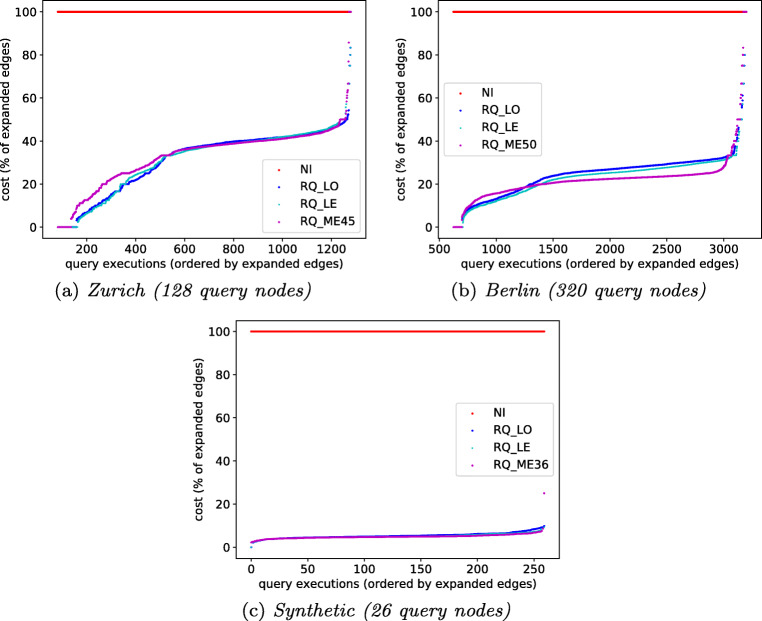
Relative number of expanded edges w.r.t. *NI* (100%)

The results for our three input graphs are shown in Fig. 8. On the y-axis we measure the relative difference of *RQ* compared to no-index baseline *NI* (horizontal red line, 100%). The data points are ordered along the x-axis by the number of expanded edges, separately for each data series. We evaluate Louvain (RQ_LO), Leiden (RQ_LE), and METIS (RQ_ME*k*). The number *k* of partitions for METIS is different for each dataset to approximately match the number of partitions that Louvain and Leiden produce for the respective setting (cf. Table 2).

In Zurich and Synthetic, all the three partitioning techniques perfom similarly for most of the data points. Leiden (RQ_LE) and Louvain (RQ_LO) perform slightly worse than METIS (RQ_ME*k*) for some of the data points on the Berlin dataset. Independently of the chosen partitioning, *RQ* clearly outperforms the baseline *NI* on all datesets: Except for a small number of outliers, the index constructed using Leiden reduces the number of expanded edges by at least 55*%* for Zurich, 70*%* for Berlin, and 90*%* for Synthetic.

#### Which Partitioning to Choose?

We summarize our experimental findings and evaluate the different partitioning techniques.

Leiden outperforms Louvain with respect to the index size. Given a similar number of partitions as Leiden and Louvain, (1) METIS produces more balanced partition sizes, but the partitions have more border nodes on average, and also (2) the index size (in particular the number of connections that dominates the index size) is smaller for Leiden than for METIS.

In terms of query performance, METIS is competitive with the other approaches and in some cases slightly outperforms them. The catch is that the performance of METIS depends on the parameter *k*, which is hard to choose without running experiments similar to the ones in our tests. The parameters *k* so determined may not be valid for other networks or query loads.

Overall we suggest to use the Leiden partitioning in conjunction with our *RQ* index.

### *RQ* vs. Competitors

In this section we compare *RQ* with its competitors *SP* and *NI* . We use *RQ* with Leiden partitioning (as we suggest in Section 7.4.4) for all experiments in this section.

#### Index Size

We compare the index size of *RQ* (with Leiden partitioning) to the index sizes of its competitors. Although *NI* does not require precomputation, the input graph has to be kept in memory. In Table 5, we compare the index sizes (*RQ* , *SP* ) to the input graph size (*NI* ).

**Table 5:** Index (RQ using Leiden partitioning)

Dataset	Algorithm	#Nodes	#Edges	#Conn
Zurich	RQ	406	3,733	425,621
	*SP*	2,508	313,500	69,464,152
	*NI*	2,508	5,630	555,713
Berlin	RQ	1,732	44,643	2,717,979
	*SP*	12,984	8,426,616	874,897,430
	*NI*	12,984	34,791	1,348,070
Synthetic	RQ	8,122	163,449	13,646,302
	*SP*	145,188	1,053,919,692	225,337,275,212
	*NI*	145,188	433,272	31,042,468

*RQ* and *SP* precompute certain shortest paths and build an index structure that is sufficient to answer reachability queries. If the index of *SP* is stored as a graph, the index nodes are identical to the input graph nodes (POIs are a subset of the input graph nodes), the number of index edges is computed as #Nodes × #POIs from the number of input graph nodes and POIs (an edge is inserted between every node and every POI), and the number of index connections results from computing a shortest path at every departure time to every POI. For *RQ* the index size depends on the position of the border nodes. We randomly pick 5% of the input graph nodes as POIs and show the average values for #Nodes, #Edges, and #Conn over five samples.

The index size of *RQ* is always smaller than that of *SP* (up to four orders of magnitude in the Synthetic graph). *RQ* is also significantly smaller than the original *Zurich* and *Synthetic* graphs (*NI* ). Although *RQ* has significantly fewer nodes and edges than the original graph for *Berlin*, the number of connections is higher. This is caused by the sparsity of *Berlin* (cf. Section 4.5). Finally, #Connections is the number of edge connections stored. For *RQ* , we list the absolute number of connections after the compaction (cf. Section 4.6). The reduction rate of compaction is high: 74% for *Zurich* and *Berlin*, and 73% for *Synthetic*.

Table 6 shows the runtime to build our *RQ* index. The shortest path computations are executed in parallel on 20 cores. For Zurich, the index builds in less than 2 min for Louvain, Leiden, and METIS. All runtimes for Berlin are below 30 min. For Synthetic, the index construction time for METIS is only 7 min with 36 partitions, but 12 hrs with 44 partitions. The high construction time for METIS with 44 partitions is due to the poor partitioning and the resulting high number of connections that require many shortest path computations (before compaction, there are 292,883,908 connections, each resulting from one shortest path computation).

**Table 6:** Index construction time

Dataset	Algorithm	Time (min)
Zurich	RQ_LO	0.7
	RQ_LE	0.7
	RQ_ME45	1.1
Berlin	RQ_LO	26.3
	RQ_LE	20.2
	RQ_ME51	29.1
Synthetic	RQ_LO	48.4
	RQ_LE	25.6
	RQ_ME36	7.2
	RQ_ME44	720.4

#### Performance of Query Answering

**Fig. 9:**
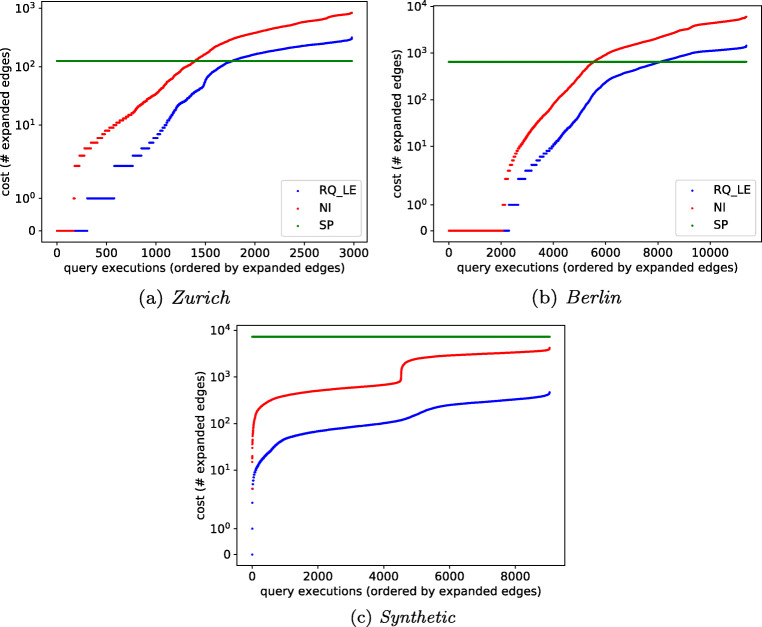
Number of expanded edges (y-axis in log scale)

To evaluate the efficiency, we compare the number of edges that each algorithm has to process in order to find all reachable POIs (Fig. 9).

In this experiment, we use our *RQ* index constructed using one of the samples discussed in Section 7.4.2. One data point in the figure (scatter plot) is a single reachability query. Data points are sorted along the x-axis by the number of expanded edges. We execute one reachability query starting at every border node in our index. We do so at five different start times (8:00, 12:00, 16:00, 18:00, 22:00) and for two time budgets (60 and 120 minutes). Thus, the number of data points is 10 ×#Border nodes. The budgets are large enough to force *RQ* to traverse multiple edges. Since the edge costs of large cells in the *RQ* index are often above 15 minutes (and above 30 minutes in about half of the cases), budgets near these values provide little insight. Since SP precomputes the path to each POI, it always evaluates one edge per POI. This is a lower bound on the cost of any point-to-point index. Although the index of SP is orders of magnitude larger, RQ expands significantly fewer edges for many of the data points. We observe the largest differences for the budget of 120 min. On Synthetic, the number of edges expanded by RQ is up to three orders of magnitude lower than that of SP, and it is up to one order of magnitude lower than that of NI. RQ always expands fewer edges than NI. Values equal to zero indicate that an algorithm cannot expand due to high connection costs.

**Fig. 10:**
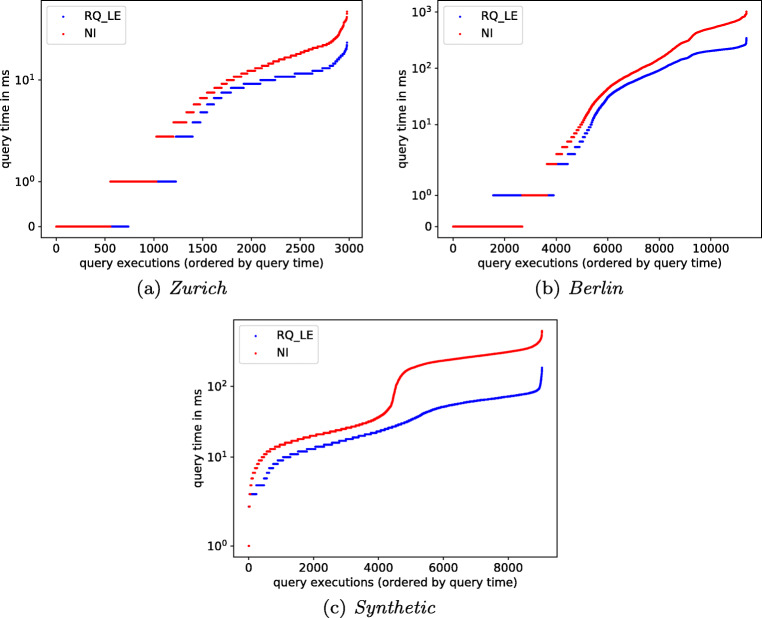
Query time in ms (y-axis in log scale)

In Fig. 10 we compare the query times of *RQ* and *NI* . We measure at the granularity of milliseconds, which results in runtime zero for very short query executions. As expected from the lower number of expanded edges, *RQ* outperforms *NI* on almost all data points across the three datasets.

Overall, our experiments show that, despite its small size, *RQ* substantially reduces the number of expanded edges, and therefore speeds up reachability queries in public transport networks.

## Conclusion

The paper offers improved support for reachability queries in temporal graphs that retrieve all reachable points of interest (POIs) from a given query node at a specific start time within a given time budget. We observe that current solutions do not scale to large networks (solutions based on Dijkstra’s algorithm without a pre-computed index) or to networks with many POIs (solutions based on an index for single-path queries that must be executed for each POI separately). We propose a solution based on a novel access structure, the reachability index. This index partitions the original temporal graph into cells, thus enabling us to expand the graph cell by cell rather than edge by edge. We evaluate different graph partitioning techniques and study the effect on index size and query performance. Our empirical evaluations suggest that our technique is both effective and efficient.

## Footnotes

1 Indexing very small disconnected components may not be useful since a reachability query expands at most the edges within the disconnected component regardless of the query parameters.

2 https://github.com/taynaud/python-louvain

3 https://github.com/vtraag/leidenalg

4 https://github.com/inducer/pymetis

5 http://glaros.dtc.umn.edu/gkhome/metis/metis/overview
